# Macrophage depletion lowers blood pressure and reduces renal fibrosis progression in existing hypertension mice model

**DOI:** 10.1016/j.jphyss.2025.100049

**Published:** 2025-10-27

**Authors:** Joseph Kasyoki Peter, Ryusuke Umene, Chia-Hsien Wu, Yasuna Nakamura, Norito Washimine, Ryoko Yamamoto, Denis Muriuki, Caroline Ngugi, Kavoo Linge, Joseph K. Kweri, Tsuyoshi Inoue

**Affiliations:** a Department of Physiology of Visceral Function and Body Fluid, Graduate School of Biomedical Sciences, Nagasaki University, Japan; b Department of Medical Physiology, School of Medicine, Jomo Kenyatta University of Agriculture and Technology, Kenya; c Department of Nephrology, Graduate School of Biomedical Sciences, Nagasaki University, Japan; d Department of Medical Microbiology, School of Biomedical Sciences, Jomo Kenyatta University of Agriculture and Technology, Kenya; e Department of Human Anatomy, School of Medicine, Jomo Kenyatta University of Agriculture and Technology, Kenya; f Department of Clinical Medicine, School of Medicine, Jomo Kenyatta University of Agriculture and Technology, Kenya

**Keywords:** Hypertension, Immune system, Renal Macrophages, Renal fibrosis

## Abstract

Uncontrolled hypertension is a global health issue with 40 % of hypertensive patients not achieving blood pressure control with current therapies. Previously, we demonstrated renal macrophage infiltration during hypertension development with macrophage depletion leading to reduced blood pressure and renal fibrosis. However, the effect of macrophage depletion in existing hypertension has not been evaluated. We induced hypertension in mice then depleted macrophages and assessed blood pressure and renal fibrosis. Separately induced hypertension and assessed renal macrophage population and fibrosis early in hypertension. Results showed increased renal macrophage, *Acta2* early in hypertension development. Macrophage depletion led to reduced blood pressure in the hypertensive mice, decreased kidney *Col1a1, Acta2, Col3a1* and *Fn1*. This study shows that renal macrophage infiltration and fibrosis begin early in hypertension development and depleting macrophages in hypertension reduces blood pressure and suppress renal fibrosis. This shows macrophages are a potential target in treatment of hypertension.

## Highlights

• Macrophage depletion lowers blood pressure in pre-existing hypertension.
• Renal macrophages infiltrate early in hypertension development.
• Macrophage depletion reduces kidney fibrosis progression in hypertension.
• Renal fibrosis begins in the early stages of hypertension.
• Targeting macrophages may offer new hypertension treatments.

## Graphical abstract

**Figure:**
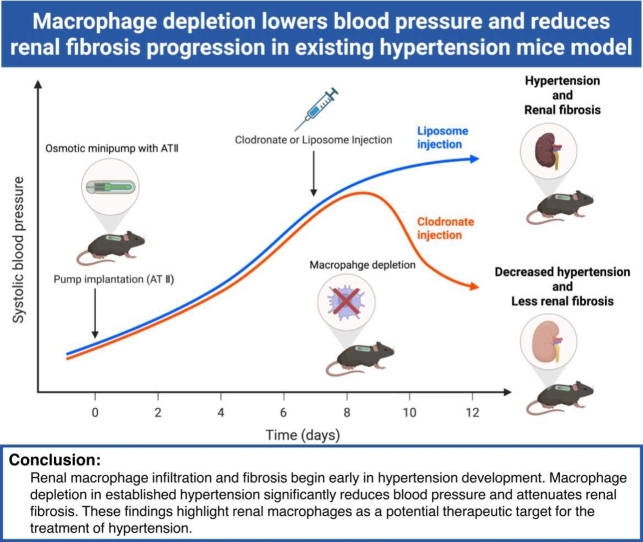

## Introduction

Hypertension is of great public health interest globally, secondary to the diseases’ implications on the health and economy of the affected individual [1]. In the last five decades, there has been major advances in hypertensive therapies; unfortunately, despite these advances, about half of hypertensive patients do not achieve blood pressure control [2], [3], [4]. Globally, suboptimal blood pressure control is among the leading attributable risk factors for death [3], [5], [6]. The existing anti-hypertensive therapies mainly target the physiological systems that regulate blood pressure including the sympathetic nervous system (SNS) and the renin angiotensin aldosterone system (RAAS) to reduce blood pressure [7], [8]. Although drugs such as ACE inhibitors and ARBs are effective and widely used, the high number of hypertensive patients fail to achieve adequate blood pressure control or remain at high risk of cardiovascular and renal complications. While agents such as angiotensin receptor–neprilysin inhibitors (ARNIs) or newer mineralocorticoid receptor antagonists (MRAs) have recently been introduced, no new class of antihypertensive drugs have been developed in the last twenty years [8]. Thus, targeting a novel physiological system for anti-hypertensive therapy is a promising approach for developing new antihypertensive drugs.

Recent studies have shown that the immune system plays a significant role in the initiation and maintenance of hypertension [9], [10], [11] making the immune system a probable target in new hypertensive therapies. Immune cells have been reported to infiltrate blood pressure regulating organs leading to hypertension [12]. We and others have previously reported macrophages involvement in the development of hypertension and showed macrophage depletion to blunt hypertension development [9], [13]. These studies reported macrophages to accumulate in the kidneys in hypertensive states and cause increase in renal fibrosis and demonstrated macrophage depletion to suppress the development of these renal changes. These findings proposed a preventive effect of macrophage depletion on hypertension, but the interventional effect in existing hypertension is not clear.

The current study explored renal macrophages and renal fibrosis in the early hypertensive development stages and explored the effect of macrophage depletion on blood pressure and renal fibrosis in existing hypertension.

## Materials and methods

### Research animals and blood pressure measurement

Male C57BL/6 J mice (8–10 weeks, 20–25 g) were purchased from Clea Japan. Inc. (Tokyo, Japan). All the animals were housed at the Research Center for Biomedical Models and Animal Welfare, Graduate School of Biomedical Sciences, Nagasaki University. Standard animal husbandry was followed with the mice fed on regular mice pellets, the rooms temperature and humidity controlled and with 12:12 h dark light cycle. All the experimental protocols were approved by the Animal Care and Use Committee of Nagasaki University, Japan. Blood pressure was measured every other day in the morning using MK-2000ST NP-NIBP monitor (Muromachi Kikai Co., Ltd, Tokyo, Japan), tail plethysmography. Mice were acclimatized to blood pressure measurement for one week before hypertension induction.

### Macrophage depletion in existing hypertension model

Hypertension was induced by Ang II (1000 ng/kg/min, Sigma-Aldrich, Co. St. Louis, USA) via subcutaneously implanted micro-Osmotic pump (Model 1002, Alzet, Cupertino, CA) under anesthesia (medetomidine hydrochloride 0.3 mg/kg, midazolam 4 mg/kg, butorphanol 5 mg/kg, i.p.). After implantation, the incision was sutured, and anesthesia was reversed with atipamezole (0.5 mg/kg, i.p.). Macrophage depletion was achieved with i.p. injection of liposome-encapsulated clodronate (10 mg/kg, Xygieia Bioscience, Osaka, Japan), 7 days after the pump implantation. Liposome was administered in the control groups. Mice were randomized into four groups: (1) Ang II + clodronate, (2) Ang II + liposome, (3) Vehicle pump + clodronate, (4) Vehicle pump + liposome.

In a separate set of experiments, macrophage depletion was also achieved using an anti-CSF1R neutralizing antibody. Mice received intraperitoneal injection of InVivoMAb anti-mouse CSF1R (CD115) (0.4 mg/mouse, Cat. BE0213, Bio X Cell) 7 days after pump implantation. Control mice were administered InVivoMAb rat IgG2b isotype control (Cat. BE0090, Bio X Cell) under the same schedule.

### Hypertension seven-day model

Six mice were grouped into two groups: 1) Ang II and saline intake, 2) Empty pump and saline intake. BP was monitored for one week after induction of hypertension.

### Kidney tissue flow cytometry leukocytes analysis

Kidney tissue digestion and flow cytometry leukocyte analysis were done as previously described by [9]. Briefly, mouse kidney tissue was digested with collagenase II (33 μg/ml, Worthington Biochemical Corp, Lakewood, NJ) for 20 min at 37°C, filtered through 70 μm and 40 μm strainers, centrifuged (500 g, 4°C, 10 min), and resuspended with Flow Cytometry Staining Buffer (Thermofisher Scientific). Cells were incubated with the following antibodies at 4°C for 30 min: anti-mouse CD45-APC (104, Biolegend), B220-PECy7 (RA3–6B2, eBioscience), CD3e-APC eFluor 780 (17A2, eBioscience), CD4-FITC (GK1.5, Biolegend), CD8a-eFluor 450 (53–6.7, eBioscience), CD11b-Brilliant Violet 421 (M1/70, Biolegend), CD11c-FITC (N418, Biolegend), F4/80-PE (BM8, Biolegend) and MHC Class II-eFluor 506 (NIMR-4, eBioscience). Dead cells were excluded using 7-AAD (Thermofisher Scientific). Leukocytes were identified as 7-AAD^−^ CD45^+^ cells. T (CD3^+^) and B (B220^+^) cells were distinguished, with CD4^+^ and CD8^+^ subsets in T cells. Macrophages were gated as CD11b^+^ F4/80^+^ cells, excluding MHC II^+^ CD11c^+^ cells from CD3^−^ B220^−^ cells. Kidney gating strategies were shown in the Supplementary Figure S1. Gating was determined using fluorescent minus 1 control samples. Data were acquired on Attune NxT Flow Cytometer (A24860; Thermofisher Scientific) and analyzed by FlowJo software 10.0 (FlowJo, LLC). The compositions of immune cell subsets were expressed in absolute terms (cells per 10⁶ kidney cells).

### Kidney histology

Kidney histology and immunohistochemistry were performed as previously described by [9]. For histological assessment of kidney fibrosis, trichrome staining was conducted on fixed and sectioned kidney tissues, with fibrosis quantified using ImageJ Ver 1.54 g, where blue staining indicated fibrotic areas. For immunohistochemistry, kidney sections were deparaffinized, subjected to antigen retrieval, and incubated overnight with a Rat anti-mouse F4/80 monoclonal antibody (MCA497R, BIO-RAD, Hercules, CA, USA) in a rat IgG blocking solution. Visualization was achieved using DAB (Maravai Inc., San Diego, CA, USA), followed by counterstaining with Hematoxylin. The F4/80-positive area was quantified using ImageJ Ver 1.54 g; Java 1.8.0_345 software.

### RNA extraction and quantitative real-time PCR (qPCR)

Kidney RNA extraction and real time qPCR was done as previously described by [9]. Specifically, kidney RNA was extracted using FastGene RNA Basic Kit (FG-80006; NIPPON Genetics, Tokyo, Japan), and reverse transcribed to cDNA using PrimeScript RT Master Mix (RR036A; Takara Bio, Shiga, Japan). Relative gene expression was measured by qPCR using the iTAC Universal SYBR Green Supermix (1725121; Bio-Rad, Tokyo, Japan). Relative gene expression was calculated using ∆∆Ct method. Primer sequences are indicated in Supplementary Table.

### Statistical analysis

Data were analyzed by one way and two-way analysis of variance (ANOVA), followed by Tukey post-hoc test. P < 0.05 was considered as significant. All values are presented as ±SEM. Data was analyzed using GraphPad Prism version 9.

## Results

### Macrophage depletion in existing hypertension lowered blood pressure

**Fig. 1:**
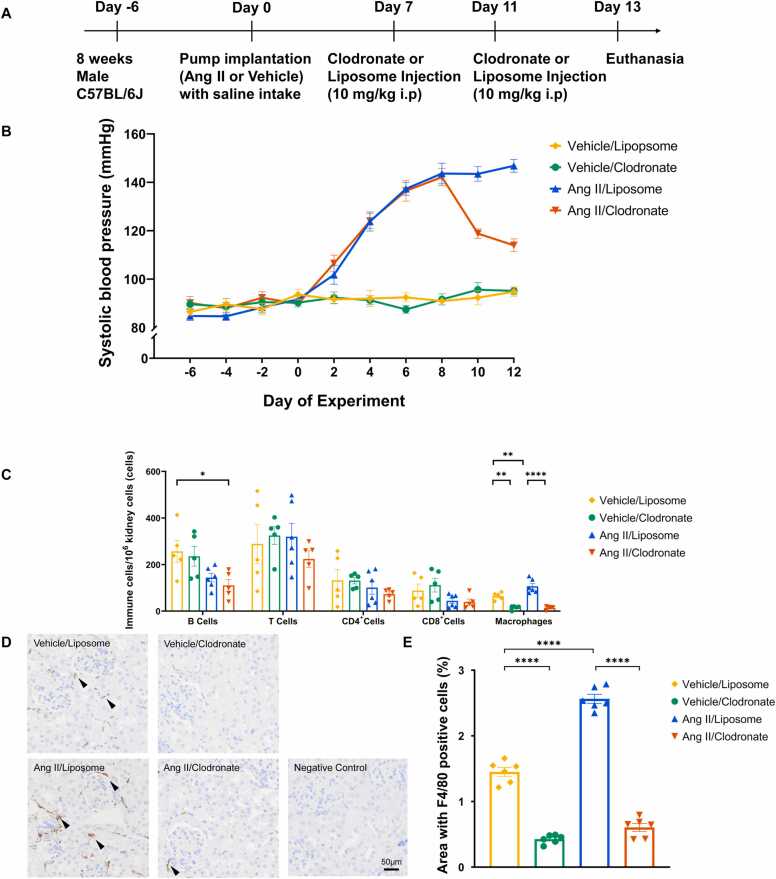
Macrophage depletion lowered blood pressure in existing hypertension, (A) Experiment timeline for macrophage depletion. (B) Blood pressure changes in different groups. Ang II administration significantly increased blood pressure up to day seven, followed by a significant reduction in existing hypertension in the Ang II/Clodronate group with macrophage depletion. (C) Flow cytometry analysis showing kidney leukocytes. (D) Kidney immunohistochemistry F4/80 expression representative images. Macrophages were effectively depleted in the clodronate treated groups compared to the liposome treated groups. Arrowheads point to F4/80 positive areas. Scale bar = 50 µm. **(E)** Quantification of renal F4/80 positive cells. F4/80 positive cells were significantly reduced in the clodronate treated groups compared to the controls. Brown color shows F4/80 positive areas. Data expressed as mean ±SEM. n = 6, **** *P* < 0.0001, ** *P* < 0.01, * *P* < 0.05.

The groups receiving Ang II had significant elevation of blood pressure from day four to day seven when compared to the control groups (*p* < 0.0001). After macrophage depletion on the seventh day, there was a significant reduction of blood pressure in the macrophage-depleted group (Ang II/Clodronate) when compared to the Ang II/liposome group (*p* < 0.0001) (Fig. 1A and B).

Similarly, in the neutralizing antibody experiments, mice treated with anti-CSF1R antibody (Ang II/CSF1R Ab) showed a significant attenuation of Ang II–induced blood pressure elevation compared with the Ang II/isotype control group (*p* < 0.001), supporting the role of macrophage depletion in suppressing hypertension (Supplementary Figure S2A and B).

### Liposome encapsulated clodronate effectively depleted renal macrophages

Liposome encapsulated clodronate has been shown to effectively deplete renal macrophages [9], [13]. Flow cytometry showed a significant decrease in renal macrophages in depletion groups compared to the control groups (*p* < 0.001) (Fig. 1C). Macrophage depletion was further confirmed by kidney F4/80 antibody immunohistochemistry (IHC) (Fig. 1D). Macrophage IHC quantification showed significant macrophage reduction in the depletion groups compared to the control groups (*p* < 0.0001) (Fig. 1E).

#### Macrophage depletion attenuated renal fibrosis in existing hypertensive model

**Fig. 2:**
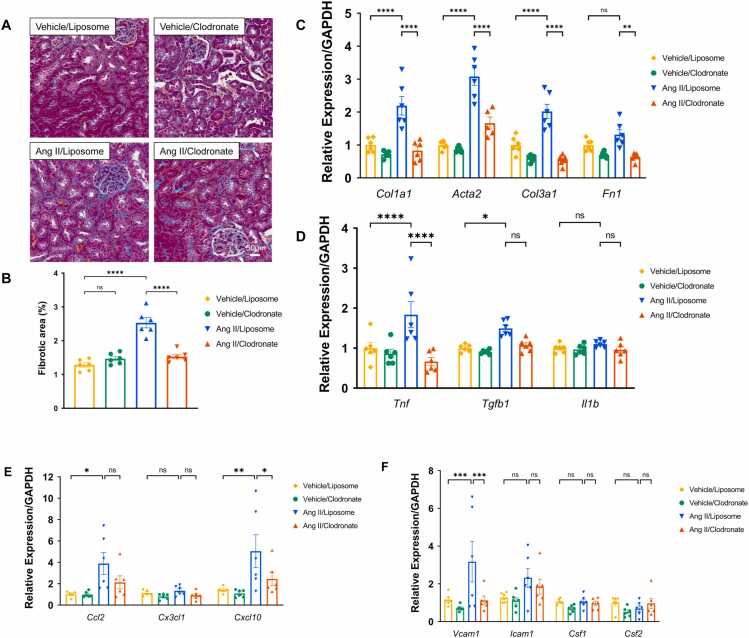
**Macrophage depletion attenuates renal fibrosis and inflammation in existing hypertension, (A)** Kidney trichrome staining representative images. Macrophage depletion in existing hypertension reduced renal fibrosis. Scale bar = 50 µm. **(B)** Quantification of renal trichome-positive staining areas. Macrophage depletion in existing hypertension significantly reduced the percentage of renal fibrotic area compared to the Ang II/liposome group. **(C)** Relative expression of fibrosis related genes (*Col1a1, Acta2, Col3a1* and *Fn1*) in the kidney. Macrophage depletion in existing hypertension significantly reduced *Col1a1, Acta2, Col3a1* and *Fn1* in the Ang II/clodronate group compared to the Ang II/Liposome group. **(D)** Relative expression of inflammatory markers (TNF⍺, TGF-β1 and IL-1β) in the kidney. Macrophage depletion in existing hypertension significantly reduced TNF⍺ expression in the kidney in the Ang II/clodronate group compared to Ang II/Liposome group. Expression of TGF-β1 and IL-1β tend to decline in the Ang II/clodronate group compared to the Ang II/Liposome group. Data expressed as mean ±SEM. n = 6, **** *P* < 0.0001, ** *P* < 0.01, * *P* < 0.05.

Kidney trichrome staining showed a significant reduction of the fibrotic areas in the Ang II clodronate group compared to the Ang II group not depleted of macrophages (*p* < 0.0001) (Fig. 2A and B). Kidney *Col1a1*, *Acta2*, *Col 3a1* (*p* < 0.0001) and *Fn1* (p < 0.01) relative expression level was significantly reduced in the Ang II-Clodronate group compared to the Ang II-liposome group (Fig. 2C).

### Macrophage Depletion lowers renal inflammation in existing hypertension

Kidney *Tnf* relative expression levels were significantly reduced (*p* < 0.0001) in the Ang II-clodronate group compared to the Ang II-liposome group. Kidney *Tgfb1* relative expression levels tend to decline in the Ang II-clodronate group compared to the Ang II-liposome group **(**Fig. 2**D).**

#### Macrophage depletion altered the expression of chemokines and adhesion molecules associated with macrophage recruitment

To explore the molecular mechanisms underlying macrophage recruitment during Ang II–induced hypertension, we examined the renal expression of chemokines, adhesion molecules, and growth factors implicated in monocyte/macrophage infiltration. qPCR analysis revealed that renal expression of *Ccl2* (MCP-1) and *Cxcl10* was markedly increased in the Ang II/liposome group, consistent with enhanced monocyte recruitment under hypertensive conditions (Fig. 2**E**). In contrast, these chemokines were significantly reduced in the macrophage-depleted group (Ang II/Clodronate), suggesting that macrophages amplify local inflammation by sustaining chemokine production. Among the adhesion molecules and colony-stimulating factors analyzed, *Vcam1* expression was significantly upregulated in response to Ang II but was markedly attenuated in the macrophage-depleted group. In contrast, *Icam1*, *Csf1* (M-CSF), and *Csf2* (GM-CSF) showed no significant changes between groups (Fig. 2**F**). These results suggest that macrophage depletion suppresses key chemokine and VCAM1-dependent monocyte adhesion molecule pathways, rather than local macrophage proliferation or differentiation driven by CSF1/CSF2 signaling, that mediate monocyte recruitment to the kidney during hypertension.

#### Renal macrophages elevate early in Ang II induced hypertension development

**Fig. 3:**
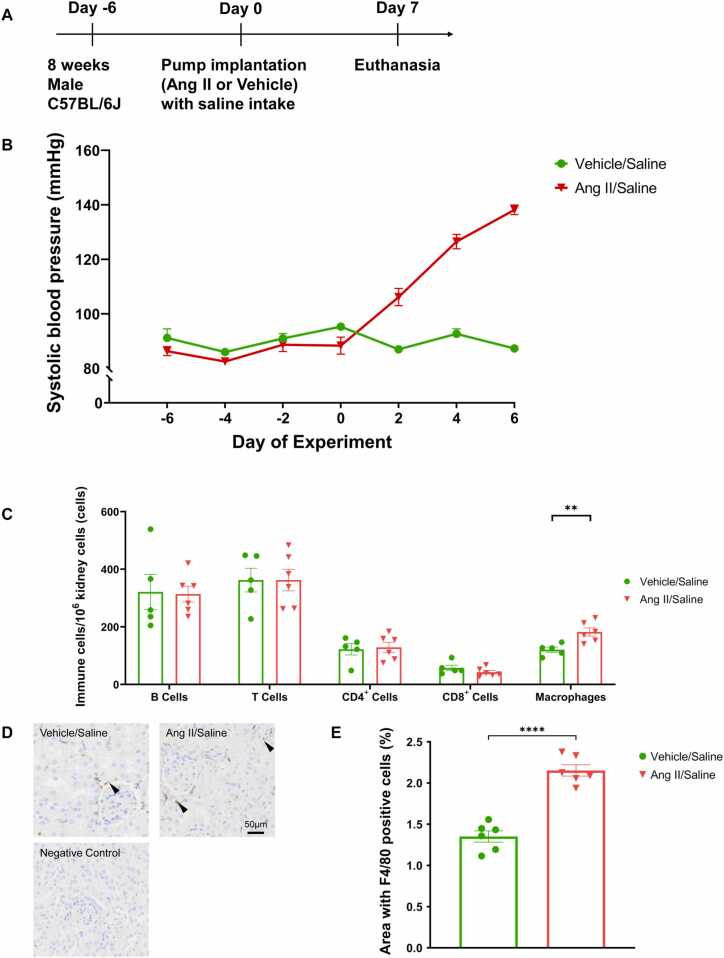
**Early effects of hypertension on renal macrophages, (A)** Experimental timeline for early effects of hypertension. **(B)** Ang II significantly elevated blood pressure in the Ang II group compared to the control group by day seven. **(C)** Flow cytometry analysis showing kidney leukocytes. Seven days after hypertension induction, the renal macrophages was significantly increased in the Ang II group compared to the control group. **(D)** Kidney immunohistochemistry F4/80 expression representative images. Ang II caused early significant macrophage increase in renal macrophages compared to the control group. Arrowheads point to F4/80 positive areas. Scale bar = 50 µm. **(E)** Quantification of renal F4/80 positive cells. F4/80 positive cells were significantly increased in Ang II group compared to the control. Brown color shows F4/80 positive areas. Data expressed as mean ±SEM. n = 6, **** *P* < 0.0001, ** *P* < 0.01.

The Ang II salt group had a significant elevation of blood pressure from day two of Ang II administration and had reached hypertensive levels by day seven **(**Fig. 3**A and B)**. Kidney flow cytometry showed a significant macrophage increase in the Ang II salt group (*p* < 0.01) when compared to the control group **(**Fig. 3**C)**. Kidney F4/80 antibody immunohistochemistry confirmed macrophage population increase in the Ang II salt group **(**Fig. 3**D)** and the quantification of macrophages showed macrophage population increased significantly in the Ang II group by day seven of Ang II administration **(**Fig. 3**E)**.

### Renal fibrosis starts to develop early in hypertension

**Fig. 4:**
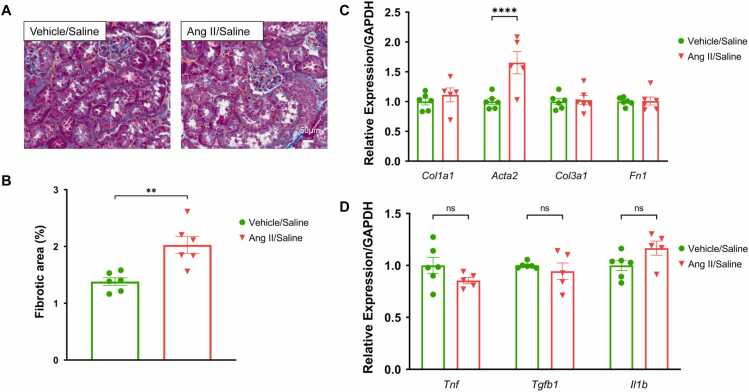
**Early effects of hypertension on renal fibrosis and inflammation, (A)** Kidney trichrome staining representative images. The Ang II group increased collagen positive areas compared to the control group. Scale bar = 50 µm. **(B)** Quantification of renal trichome-positive staining areas. The Ang II group significantly increased percentage of renal fibrotic areas (blue stained) compared to the control group. **(C)** Relative expression of fibrosis related genes (*Col1a1, Acta2, Col3a1* and *Fn1*) in the kidney. *Acta2* expression was significantly increased in the Ang II group early in hypertension development compared to the control group. However, *Col1a1 Col3a1* and *Fn1* relative expression did not increased in the early phases of hypertension. **(D)** Relative expression of inflammatory markers (TNF⍺, TGF-β1 and IL-1β) in the kidney. At day seven of hypertension, no inflammatory marker level increased in the Ang II group compared to the control group. Data expressed as mean ±SEM. n = 6, **** *P* < 0.0001, ** *P* < 0.01.

Kidney tissue trichrome staining and quantification of collagen-stained areas (blue interstitial areas) showed a significant increase (*p* < 0.05) of the collagen-stained positive areas in the Ang II salt group compared to the control group **(**Fig. 4**A and B)**. Examination of the gene relative expression levels of fibrosis markers *Col1a1*, *Col 3a1* and *Fn1* showed no difference between the hypertensive group and the control group while *Acta2* was significantly elevated (*p* < 0.0001) in Ang II group compared to the control **(**Fig. 4**C)**.

### Renal inflammation occurs late in hypertension

Seven days post hypertension induction, there was no difference in kidney *Tnf* and *Tgfb1* relative expression levels in the Ang II and salt group compared to the control group **(**Fig. 4**D)**.

## Discussion

Immune cells are important in protecting against pathogens. However, their chronic overactivation can lead to tissue damage and hypertension [4]. We and others recently demonstrated that macrophages accumulate in the kidneys in hypertension development and lead to renal fibrosis [9], [13], [14]. Understanding early stages of renal macrophage activation in hypertension development will help the overall understanding of hypertensive renal injury and identify therapeutic strategies to break the cycle of renal injury-immune system activation [15].

Hypertensive animal models have demonstrated that, continued immune cells accumulation in the kidneys plays a pathogenic role in the progressive nature of hypertensive renal disease [15], [16]. Immune cell activation mechanisms in hypertension also lead to the production of immune cell products such as cytokines and reactive oxygen species which in a feed forward manner leads to further blood pressure elevation [17]. Macrophage accumulation in the kidneys in hypertension development has been reported in several studies suggesting macrophages as a major player in the process of hypertension development [9], [13], [15], [18]. This makes macrophages a promising target in the development of hypertensive therapies and its related complications.

In this current study, macrophages were found to infiltrate kidneys by day seven of hypertension development, and macrophage depletion after day seven of hypertension led to a decrease in blood pressure. These results suggest that macrophage activation had already occurred in the onset of hypertension, and depleting macrophage in existing hypertension lowered the already elevated blood pressure. This signifies that interventions targeting stopping macrophage activation in existing hypertension can be promising in lowering blood pressure hence useful in the management of existing hypertension.

Myofibroblasts are collagen-producing cells involved in tissue fibrosis and are characterized by ⍺ SMA, which is the primary cell type responsible for renal fibrosis [19]. Macrophages activate myofibroblasts in fibrotic niches, leading to fibrosis [20]. Renal interstitial fibrosis has been shown to be the main complication of hypertensive nephropathy [21], [22]; therefore, the prevention and treatment of renal interstitial fibrosis could be a promising approach in managing hypertensive nephropathy. It has been demonstrated that TGF-β1 is upregulated in the glomeruli and interstitium of fibrotic kidneys, promoting renal fibrosis [23]. Furthermore, Wang et al. [24] reported that TGF-β1 induces macrophage-myofibroblast migration and collagen production. This study found that macrophage depletion in established hypertension reduces renal fibrosis and TGF-β1 levels. Acta2, a gene encoded ⍺SMA, was also found to elevate early in the development of hypertension and was reduced after macrophage depletion, indicating the early onset of renal fibrosis in hypertension and that macrophage depletion reversed renal fibrosis. Additionally, this study found that fibrotic markers Col1a1, Col3a1, and Fn1, as well as renal interstitial fibrosis, were significantly reduced following macrophage depletion in existing hypertension. To the best of our knowledge, this is the first study to show early effect of hypertension on renal immune cell and the first to show the effect of macrophage depletion on renal fibrosis and blood pressure in existing hypertension.

In the early stages of hypertension, inflammatory cytokines alter kidney blood flow and the expression of the sodium transporter, leading to sodium retention and elevated blood pressure [25]. In vitro studies have shown that increased NaCl enhances macrophages responses to lipopolysaccharide (LPS), upregulating IL-1β, IL-6 and IL-1⍺ [26]. In the current study, macrophage depletion in established hypertension significantly reduced renal TNF⍺, TGF-β1, and IL-1β levels, suggesting that renal macrophages sustain hypertension by mediating inflammation. Moreover, we demonstrate that Ang II–induced hypertension is associated with increased renal macrophage accumulation, which amplifies local inflammation through the upregulation of chemokines and adhesion molecules. The elevated expression of *Ccl2* and *Cxcl10* under Ang II stimulation indicates an enhanced monocyte recruitment response, while the reduction of these chemokines in the macrophage-depleted group suggests that macrophages actively sustain the inflammatory environment in the kidney. Interestingly, *Vcam1* expression was significantly upregulated by Ang II and attenuated by macrophage depletion, whereas *Icam1*, *Csf1*, and *Csf2* remained unchanged. This differential pattern implies that VCAM1-dependent monocyte adhesion and infiltration—rather than CSF1/CSF2-mediated local proliferation or differentiation of resident macrophages—represents the dominant mechanism driving macrophage accumulation in the hypertensive kidney. Furthermore, the observed downregulation of VCAM1 upon macrophage depletion suggests a positive feedback loop in which infiltrating macrophages promote endothelial VCAM1 expression, likely through proinflammatory cytokines such as TNF-α, thereby perpetuating leukocyte recruitment. Taken together, these findings support a model in which Ang II–induced hypertension involves a VCAM1–chemokine axis–driven recruitment and activation of macrophages, rather than local macrophage expansion via CSF pathways. This mechanism may contribute to renal inflammation, fibrosis, and impaired sodium handling, ultimately exacerbating blood pressure elevation. Therapeutically targeting monocyte recruitment or disrupting the VCAM1–chemokine axis may therefore represent a promising approach to attenuate hypertensive renal injury. Therefore, macrophage-targeted interventions in existing hypertension may reduce renal inflammation lowering blood pressure, giving hope for macrophage modulation therapies in the management of existing hypertension.

Additionally, macrophage infiltration and renal fibrosis were observed early in hypertension development, but the onset of renal inflammation is delayed. Although fibrosis usually progresses following inflammation, these results imply that early renal macrophage infiltration and renal fibrosis initially develop independently of renal inflammation, inflammation develops later and may subsequently exacerbate the renal macrophage infiltration and renal fibrosis. These results suggest macrophage modulation has great potential to emerge as a novel method in management hypertension.

Although the present study demonstrated that macrophage depletion lowered blood pressure and reduced renal inflammatory cytokines, the precise mechanism underlying the early decline in systolic blood pressure after LEC administration remains uncertain. It is plausible that suppression of RAAS activity and inflammatory mediators contributes to this effect [17], [27]; however, our current data are insufficient to draw a definitive conclusion. Other mechanisms, such as changes in renal sodium handling or vascular reactivity, may also be involved [28]. This point has been acknowledged as a limitation of the present study.

A further limitation concerns the measurement of blood pressure using tail-cuff plethysmography, which has technical difficulties in accurately determining diastolic blood pressure [29], [30] Therefore, we focused our analysis on systolic blood pressure, as diastolic values could not be reliably obtained with this method.

In addition, the use of intraperitoneal injection of liposome-encapsulated clodronate resulted in systemic macrophage depletion across multiple organs rather than kidney-specific depletion. This limitation affects the specificity of renal macrophage depletion in the present study. To refine the specificity of renal macrophage depletion, future studies should consider local administration of liposome encapsulated clodronate, such as renal subcapsular or intraparenchymal injection, to more directly establish the link between renal macrophages and blood pressure regulation. These local delivery approaches will be valuable to substantiate the renal macrophages effect on blood pressure.

## Conclusions

Our study demonstrates that renal macrophage infiltration and fibrosis begin early in hypertension development. Importantly, macrophage depletion in established hypertension significantly reduces blood pressure and attenuates renal fibrosis. These findings highlight renal macrophages as a potential therapeutic target for the treatment of hypertension.

## Funding sources

This work was supported by funds from the Japan Society for the Promotion of Science Grant-in-Aid for Scientiﬁc Research (B) (10.13039/501100001691JSPS KAKENHI grant 22H03090); 10.13039/100009619Japan Agency for Medical Research and Development (AMED PRIME JP22gm6210013); 10.13039/501100002241Japan Science and Technology Agency (JST) FOREST (Fusion Oriented Research for disruptive Science and Technology) (JPMJFR210J); MSD Life Science Foundation; 10.13039/100009666Salt Science Research Foundation (No. 1919&22C5); SRF; 10.13039/100007449Takeda Science Foundation; 10.13039/501100007263Astellas Foundation for Research on Metabolic Disorders; 10.13039/100007434Suzuken Memorial Foundation; 10.13039/100011313Tokyo Biochemical Research Foundation; Japan Kidney Association and Nippon Boehringer Ingelheim Joint Research Project; The Naito Foundation; 10.13039/501100005927Daiichi Sankyo Foundation of Life Science; The Uehara Memorial Foundation; Terumo Life Science Foundation to Tsuyoshi Inoue; the Japan Society for the Promotion of Science Grant-in-Aid for Research Activity Start-up (JSPS KAKENHI grant 24K23464); 10.13039/100007434Suzuken Memorial Foundation; the 10.13039/501100003837Ichiro Kanehara Foundation for the Promotion of Medical Sciences and Medical Care; Nagasaki University Grant for Co-creation Research to Ryusuke Umene; the Japan Society for the Promotion of Science Grant-in-Aid for Research Activity Start-up (JSPS KAKENHI grant 21K20874); the Japan Society for the Promotion of Science Grant-in-Aid for Young Scientists (JSPS KAKENHI grant 23K15251) to Yasuna Nakamura; and the Japan Society for the Promotion of Science Grant-in-Aid for Young Scientists (JSPS KAKENHI grant 22K16107 and 24K19036); 10.13039/100015060Nagasaki University Grant for Co-creation Research to Chia-Hsien Wu.

## CRediT authorship contribution statement

**Ryusuke Umene:** Writing – review & editing, Writing – original draft, Visualization, Validation, Supervision, Software, Resources, Project administration, Methodology, Investigation, Funding acquisition, Formal analysis, Data curation, Conceptualization. **Joseph Kasyoki Peter:** Writing – review & editing, Writing – original draft, Visualization, Project administration, Methodology, Investigation, Formal analysis, Data curation, Conceptualization. **Tsuyoshi Inoue:** Writing – review & editing, Writing – original draft, Supervision, Software, Resources, Project administration, Methodology, Investigation, Funding acquisition, Formal analysis, Data curation, Conceptualization. **Kweri Joseph. K.:** Writing – review & editing, Supervision. **Kavoo Linge:** Writing – review & editing, Supervision. **Caroline Ngugi:** Writing – review & editing, Supervision. **Denis Muriuki:** Writing – review & editing, Supervision. **Ryoko Yamamoto:** Visualization, Methodology, Investigation, Data curation. **Norito Washimine:** Writing – review & editing, Methodology, Investigation, Data curation, Conceptualization. **Yasuna Nakamura:** Writing – review & editing, Funding acquisition. **Chia-Hsien Wu:** Writing – review & editing, Funding acquisition.

## Author contributions

Joseph Kasyoki Peter, Ryusuke Umene, Tsuyoshi Inoue, Norito Washimine, Chia-Hsien Wu, Yasuna Nakamura and Joseph K. Kweri designed the research studies. Joseph Kasyoki Peter, Ryusuke Umene, Norito Washimine and Ryoko Yamamoto conducted the experiments, Joseph Kasyoki Peter analyzed the data and wrote the original manuscript, Ryusuke Umene, Tsuyoshi Inoue reviewed and edited the original manuscript. Denis Muriuki analyzed data. Tsuyoshi Inoue, Ryusuke Umene, Kavoo Linge, Caroline Ngugi and Joseph K. Kweri supervised the study. All the authors edited and approved the final version of the manuscript.

## Declaration of Competing Interest

The authors declare that they have no known competing financial interests or personal relationships that could have appeared to influence the work reported in this paper.

## Data Availability

The data that support the findings of this study are available from the corresponding author, upon reasonable request.

## Appendix A Supplementary material

Supplementary Figure S2: **Macrophage depletion with anti-CSF1R neutralizing antibody lowered blood pressure in existing hypertension, (A)** Experiment timeline for macrophage depletion. **(B)** Blood pressure changes in different groups. Ang II administration significantly increased blood pressure up to day seven, followed by a significant reduction in existing hypertension in the Ang II/CSF1R Ab group with macrophage depletion

## Footnotes

Appendix A Supplementary data associated with this article can be found in the online version at doi:10.1016/j.jphyss.2025.100049.
