# Supplementary material for: Macrophage depletion lowers blood pressure and reduces renal fibrosis progression in existing hypertension mice model
Source: J Physiol Sci. 2025 Oct 27;75(3):100049. doi: 10.1016/j.jphyss.2025.100049 (PMC12616062; doi:10.1016/j.jphyss.2025.100049)
Supplement: Supplementary file 1 — Supplementary Figure S2: Macrophage depletion with anti-CSF1R neutralizing antibody lowered blood pressure in existing hypertension, (A) Experiment timeline for macrophage depletion. (B) Blood pressure changes in different groups. Ang II administration significantly increased blood pressure up to day seven, followed by a significant reduction in existing hypertension in the Ang II/CSF1R Ab group with macrophage depletion [file mmc1.pptx]

## Slide 1
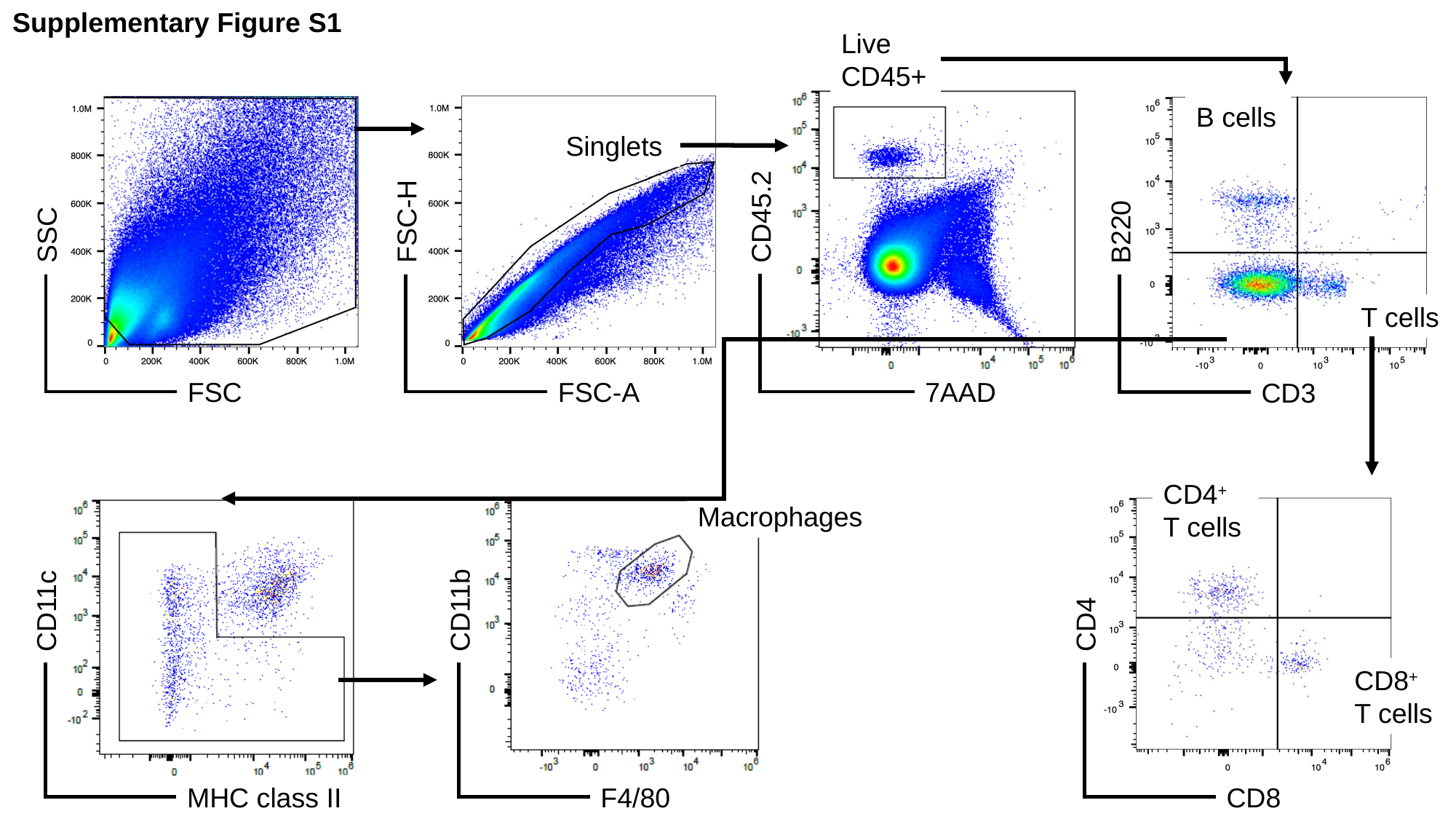

Supplementary Figure S1
Live
CD45+
B cells
Singlets
CD45.2
SSC
FSC-H
B220
T cells
FSC
FSC-A
7AAD
CD3
CD4+
T cells
Macrophages
CD11c
CD11b
CD4
CD8+
T cells
MHC class II
F4/80
CD8

## Slide 2
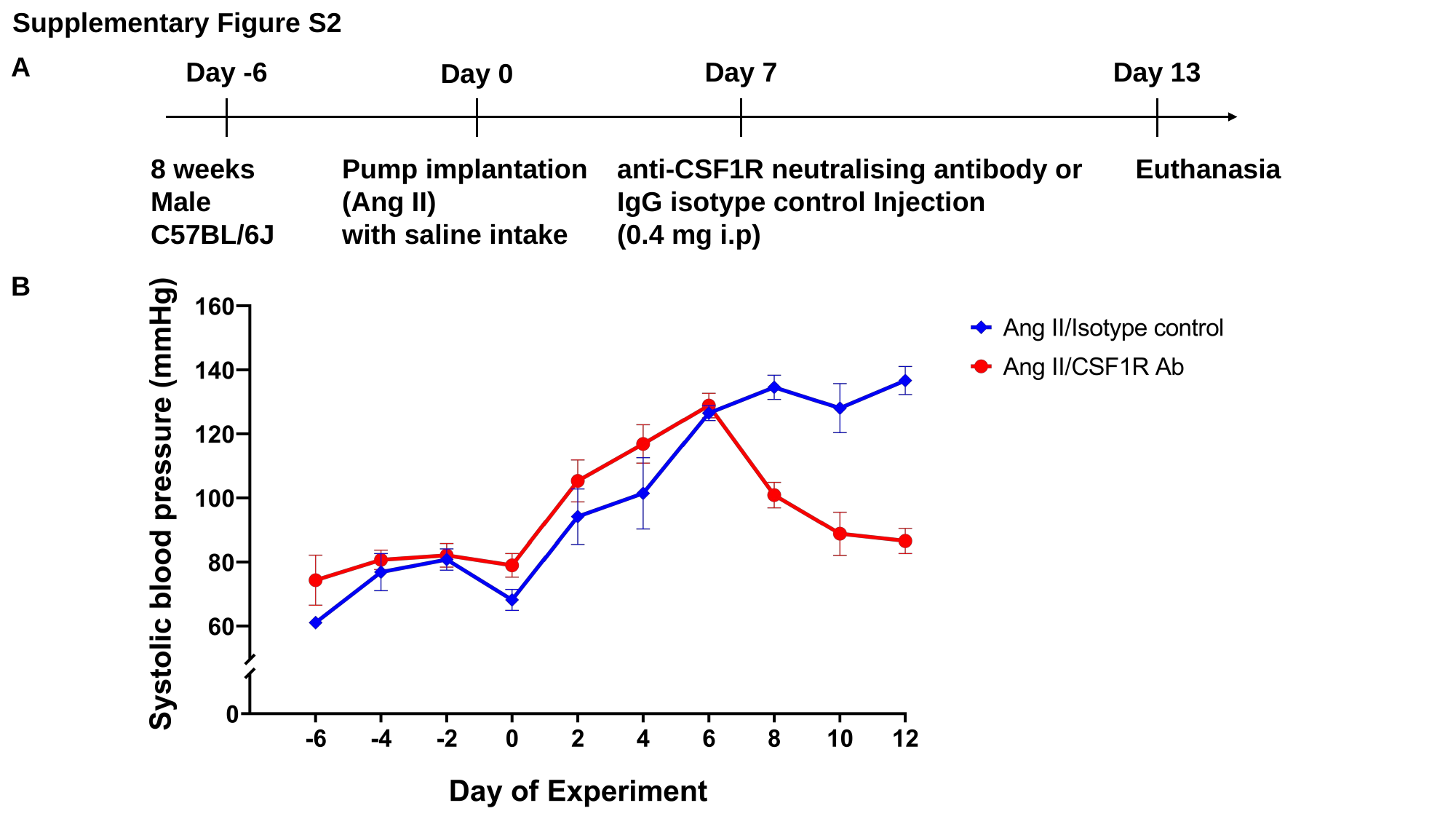

Supplementary Figure S2
A
Day -6
Day 7
Day 13
Day 0
8 weeks Male C57BL/6J
Pump implantation (Ang II)
with saline intake
anti-CSF1R neutralising antibody or IgG isotype control Injection
(0.4 mg i.p)
Euthanasia
B
