# Supplementary material for: Macrophage depletion lowers blood pressure and reduces renal fibrosis progression in existing hypertension mice model
Source: J Physiol Sci. 2025 Oct 27;75(3):100049. doi: 10.1016/j.jphyss.2025.100049 (PMC12616062; doi:10.1016/j.jphyss.2025.100049)
Supplement: Supplementary file 2 — Supplementary Table [file mmc2.docx]

**Supplementary Table: Real-time quantitative PCR Primer sequences**

*Gapdh* forward AGGTCGGTGTGAACGGATTTG

*Gapdh* reverse TGTAGACCATGTAGTTGAGGTCA

*Col1a1* forward GAGCGGAGAGTACTGGATCG

*Col1a1* reverse TACTCGAACGGGAATCCATC

*Acta2* forward ATTGTGCTGGACTCTGGAGATGGT

*Acta2* reverse TGATGTCACGGACAATCTCACGCT

*Col3a1* forward TCCTAACCAAGGCTGCAAGATGGA

*Col3a1* reverse ACCAGAATCTGTCCACCAGTGCTT

*Fn1* forward ACCAACCTTAATCCGGGCAC

*Fn1* reverse TCAGAAACTGTGGCTTGCTGG

*Tnf* forward GCCTCTTCTCATTCCTGCTTG

*Tnf* reverse CTGATGAGAGGGAGGCCATT

*Tgfb1* forward ACGTCACTGGAGTTGTACGG

*Tgfb1* reverse GGGGCTGATCCCGTTGATTT

*Il1b* forward AGCAGCATCACCTTCGCTTAG

*Il1b* reverse GTGTCCAGATATTGGCATGGG

*Ccl2* forward GACCTTAGGGCAGATGCAGT

*Ccl2* reverse AGCTGTAGTTTTTGTCACCAAGC

*Cx3cl1* forward CCGCGTTCTTCCATTTGTGT

*Cx3cl1* reverse AAGCCACTGGGATTCGTGAG

*Cxcl10* forward GATGACGGGCCAGTGAGAAT

*Cxcl10* reverse CTCAACACGTGGGCAGGATA

*Vcam1* forward TGAACCCAAACAGAGGCAGAGT

*Vcam1* reverse GGTATCCCATCACTTGAGCAGG

*Icam1* forward CAATTTCTCATGCCGCACAG

*Icam1* reverse AGCTGGAAGATCGAAAGTCCG

*Csf1* forward ACCCAGGATGAGGACAGACA

*Csf1* reverse GGGTAGTGGTGGATGTTCCC

*Csf2* forward TCACTGGCCCCATGTATAGC

*Csf2* reverse GAGTACTGGGCTCACTGCAA
